# Understanding job satisfaction and motivation among nurses in public health facilities of Ethiopia: a cross-sectional study

**DOI:** 10.1186/s12912-019-0373-8

**Published:** 2019-10-15

**Authors:** Firew Ayalew, Sharon Kibwana, Shelemo Shawula, Equlinet Misganaw, Zeine Abosse, Jos van Roosmalen, Jelle Stekelenburg, Young Mi Kim, Mihereteab Teshome, Damtew Wolde Mariam

**Affiliations:** 1 Jhpiego Ethiopia, Addis Ababa, Ethiopia; 2 Jhpiego, 1615 Thames St # 200, Baltimore, MD 21231 USA; 3 Management Sciences for Health, Addis Ababa, Ethiopia; 4 Athena Institute, VU University, Amsterdam, the Netherlands; 5 Department of Health Sciences, Global Health, University Medical Centre Groningen, University of Groningen, Groningen, the Netherlands; 6 Department of Obstetrics and Gynecology, Leeuwarden Medical Centre, Leeuwarden, the Netherlands

**Keywords:** Job satisfaction, Motivation, Nurse, Human resources management, Public health facility, Hospital, Health center, Ethiopia

## Abstract

**Background:**

Poor job conditions and limited resources are reducing job satisfaction and motivation among nurses in low-income countries, which may affect the quality of services and attrition rates. The objective of this study was to examine job satisfaction, motivation and associated factors among nurses working in the public health facilities of Ethiopia, with the aim of improving performance and productivity in the health care system.

**Methods:**

The study employed a cross-sectional two-stage cluster sampling design. From a random sample of 125 health facilities, 424 nurses were randomly selected for face-to-face interviews in all regions of Ethiopia. Nurses responded to questions about their overall job satisfaction and job conditions, including items related to intrinsic and extrinsic motivation, using a 5-point Likert scale. Multilevel analysis was performed to adjust for different clustering effects. Satisfaction levels (percent of respondents who were satisfied) were calculated for individual items, and composite mean scores (range: 1–5) were calculated for motivational factors. Adjusted odds ratios were computed to examine the association of these factors with overall job satisfaction.

**Results:**

Overall, 60.8% of nurses expressed satisfaction with their job. Composite mean scores for intrinsic and extrinsic motivational factors were 3.5 and 3.0, respectively. Job satisfaction levels were significantly higher for female nurses (65.6%, *p* = 0.04), those older than 29 years (67.8%, *p* = 0.048) and had over 10 years work experiences (68.8%, *p* = 0.007). Satisfaction with remuneration (AOR = 2.04, 95% CI = 1.36, 3.06), recognition (AOR = 2.21; 95% CI = 1.38, 3.53), professional advancement (AOR = 1.54; 95% CI = 1.06, 2.29), features of the work itself (AOR = 1.65; 95% CI = 1.20, 2.91) and nurses’ work experiences from 5 to 10 years (AOR = 0.37, 95% CI = 0.17, 0.79) were significantly associated with overall job satisfaction after controlling for other predictors.

**Conclusions:**

The study findings are signals for the Ministry of Health to strengthen the human resource management system and practices to improve nurses’ overall job satisfaction and motivation, especially among nurses with 5 to 10 years of experience on the job. Expanded recognition systems and opportunities for advancement are required to increase nurses’ job satisfaction and motivation. Equitable salary and fringe benefits are also needed to reduce their dissatisfaction with the job.

## Background

Ethiopia is the second most populous nation in Africa, with a life expectancy at birth of 65.5 years in 2016 [1]. The country has successfully scaled up multifaceted interventions in the health sector, expanded health science training institutions and universal health service coverage, and trained a massive health workforce as part of its efforts to achieve the Millennium Development Goals [2]. Remarkable achievements have been observed, including a 67% reduction in the under-five mortality rate (from 204 per 1000 live births in 1990 to 67 in 2016) [2, 3] and a 70% reduction in the maternal mortality ratio (from 1400 per 100,000 live births in 1990 to 412 in 2016) [2, 3]. Ethiopia has also made substantial progress in expanding the coverage of institutional delivery by skilled health providers from 5% in 2000 to 28% in 2016 [1, 3]. However, this increase is very low compared with neighboring countries [1]. Morbidity and mortality due to malaria, HIV/AIDS, and tuberculosis have also declined [2].

Human resources are key elements for improving the performance of health care system. Sufficient numbers of competent and motivated health workers, and adequate resources and funding are important factors to achieve the national and international health related goals [4]. The Sustainable Development Goals (SDGs) call for countries to increase the aggregate density of physicians, nurses, and midwives to 4.45 per 1000 population to achieve health-related targets by 2030 [5]. Hence, the Federal Ministry of Health (FMoH) of Ethiopia launched a 2016–2025 strategic plan for human resources for health (HRH) to guide the country’s effort to develop, recruit, deploy, motivate and retain health workers [6]. Ethiopia succeeded in doubling the density of all categories of health professionals from 0.84 to 1.63 per 1000 population from 2010 to 2016; this figure is expected to rise to 3.0 per 1000 in 2025 [6].

The nursing workforce in Ethiopia plays an important role in providing direct primary health care in remote and rural areas, as well as high quality nursing care to patients in hospitals. Most nurses hold a diploma from a three-year program at a Technical and Vocational Education Training (TVET) institution. Other nurses hold bachelor’s and master’s degrees from a university; these programs require 4 years and 2 years of study, respectively. Nurses who graduated in diploma are eligible to continue the 4 years training program at university after they provide 2 years of services in clinical settings and successfully pass certificate of competence test. Similarly, nurses who graduated in bachelor’s degree can join the 2 years masters training program after they provide 2 years of services at health facility level and pass entrance examination. Nurses are the largest health provider cadre in Ethiopia, numbering 50,604 in 2016; their number is projected to reach 127,299 in 2025 [6]. Unpublished FMoH data show that currently, 92% of the nursing workforce in Ethiopia have diploma, 3% have bachelor’s degree and 5% have master’s degree. Hence the HRH projections show that Ethiopia will need to deploy an additional 24,558 bachelor-level and 344 masters-level nurses at primary health care facilities and specialized hospitals by 2025 to meet the needs of the country’s growing population [6]. The nurse-to-population ratio was 1 per 2132 people in 2016; this is considerably less than the ratio of 1 nurse per 967 people found in the neighboring country of Kenya [7]. Maldistribution, low job satisfaction and motivation, and high attrition rates pose major challenges for nurses and other health workers to provide quality health care services in remote and rural areas of Ethiopia [2].

Two types of factors – related to intrinsic and extrinsic motivation – primarily drive overall job satisfaction. Intrinsic motivational factors include achievement, recognition for achievement, features of the work itself, responsibility and personal growth or advancement. Extrinsic motivational factors are related to the job’s context and include policies and administration, supervision, interpersonal relationships, working conditions, salary, status, security, and personal life [8]. Intrinsic motivational factors are more powerful than extrinsic motivational factors at increasing job satisfaction and improving performance [8]. In contrast, extrinsic motivational factors do not provide long-term job satisfaction, rather they prevent dissatisfaction or unhappiness on the job [8]. Various studies indicate that low job satisfaction and poor motivation are the leading causes of nurses’ attritions and turnover intentions [9–11].

A recent systematic reviews conducted globally noted that a range of factors are affecting nurses’ job satisfaction, motivation and retention. The reported diverse factors include: nurses’ empowerment at workplace, working conditions, living conditions, career development, pay and other financial and non-financial incentives [12–14]. Another review concluded that enhancing nurses’ job satisfaction, motivation and retention have positive impact on quality of health care services, improving nursing work environment and reduction of organizational costs related to recruitment and hiring of new nurses for replacement [15]. Previous studies have also shown that nurses’ socio-demographic characteristics (e.g. age, sex, education, experience) and workplace characteristics are associated with health workers’ job satisfaction and motivation [16–20].

Globally, there is no shortage of studies on nurses’ job satisfaction, motivation and retention; however, most studies are descriptive and focused on specific nurses’ job conditions at hospitals, mainly in high income countries [15]. The weaknesses of these studies limit their power to produce generalizable findings for making evidence-based decisions for nursing workforce in low income countries [15]. According to the World Health Organization (WHO), additional research is needed to understand existing health worker retention schemes and HRH gaps in low-income countries to guide evidence-based policies to achieve the SDGs and universal health coverage [5, 21]. This is certainly the case in Ethiopia, where previous small-scale studies have focused on specific districts, have largely been limited to hospital settings, and have employed a small number of job-related items; they have found mixed results regarding nurses’ job satisfaction, motivation, and retention [22–29]. Moreover, the FMoH has identified many national-level HRH evidence gaps, including for nurses’ job satisfaction, motivation and retention [2, 6].

The objective of this study was to examine job satisfaction, motivation, and associated factors among nurses working in the public health facilities of Ethiopia, with the aim of improving performance and productivity in the health care system. The specific research questions were: 1) What is the overall level of job satisfaction among nurses in Ethiopia, and how are nurses’ characteristics and job conditions associated with overall job satisfaction? 2) What are nurses’ perceptions of specific job conditions, and do they vary by nurses’ characteristics?

## Methods

### Study design, setting and sampling

A cross-sectional two-stage cluster sampling design was used. First, a random sample of public health facilities in all 11 regions of the country was selected, and then nurses were randomly selected at each facility in the sample.

In 2013, 45,509 nurses were serving in 3372 public health facilities in Ethiopia, including 127 hospitals and 3245 health centers [30, 31]. A nationally representative sample size was calculated with the assumptions of 95% level of statistical confidence, 50% job satisfaction level (which was set at this level because of the lack of any prior national-level estimates of job satisfaction), 5% margin of error, and a default value of design effect 1.2, where there was no estimate found on design effect [30, 32]. This yielded a sample size of 500 nurses, after adjusting for a 10% non-response rate.

The HRH strategic plan indicated that there were a minimum of five nurses stationed at each public health facility during the study period [6]. Based on a MEASURE Evaluation recommendation regarding sampling of health care providers per facility [32], we decided to invite four nurses at each of 125 health facilities to participate in the study in order to achieve the total sample of 500 nurses. The four nurses were selected at random from all those assigned to the facility. If fewer than four nurses were found at a facility during the data collection period, additional nurses were selected at the next facility.

The 125 health facilities in the sample were allocated proportionally to facility type (9 hospitals and 116 health centers) to each region to obtain heterogeneous information and perform subgroup analysis. Using a list of all health facilities in the country obtained from the FMoH, we then randomly selected 116 health centers from a total of 3245 health centers and 9 hospitals from a total of 127 hospitals.

### Data collection instruments

The study used a structured questionnaire that was developed by Management Sciences for Health, an international non-governmental organization, and piloted in Uganda [33]. The FMoH and study team members reviewed and adapted the questionnaire to fit the Ethiopian context. The questionnaire was adapted to include additional background information related to the Ethiopian health care system and nurse’s characteristics. For example, variables such as nurses’ years of service obligation under the compulsory health service scheme and their socio-demographic characteristics (including family size, marital status, and work experience) were included. The questionnaire contained sections on job satisfaction, motivation and intention to leave the job. We used the job satisfaction and motivation sections to understand the levels and associated factors of job satisfaction and motivation. These included 34 questions on job conditions, a global rating of job satisfaction (“Considering everything I am satisfied with my job”) and 13 questions on nurses’ socio-demographic characteristics and employment (e.g., sex, age, years of service, educational qualifications, type of facility, and region). A 5-point Likert scale was used to respond to all questions (1 = strongly disagree, 2 = disagree, 3 = neutral, 4 = agree, and 5 = strongly agree). The questionnaire was translated from English into Amharic and piloted in one public hospital with 10 health workers, including nurses, to check for clarity and flow of the questions and to ensure that respondents understood the questions. Minor changes were made to the questionnaire after the pilot test.

### Data collection procedures

Data were collected in June 2014. Data collectors and supervisors were health professionals who had experience in data collection. A total of 24 data collectors and 11 supervisors were trained for 3 days on ethics, interview techniques, and data quality. During visits to each health facility, data collectors explained the purpose of the study to the facility manager, asked for a list of all nurses at that facility, and randomly selected four nurses for face-to-face interviews. Data collectors obtained verbal consent from nurses after informing them about the nature of the study and that their participation was voluntary. Then they scheduled individual interviews to avoid disruption of patient care. Data collectors conducted interviews in a private room to maintain confidentiality of nurses’ responses and protect their identity (name and identification number). Supervisors checked completed questionnaires in the field to ensure data quality.

### Data management and analysis

Data were cleaned and entered into Epi-Info 7, and then exported to STATA 14.1 for statistical analysis. Further data cleaning was performed by reviewing the results of frequencies and percentages to examine data inconsistencies, missing cases and outliers.

Multilevel analysis was used to account for variations in job satisfaction and job conditions among nurses and at the health facility level. Two level nested structures were employed. The first level (randomly selected nurses) was nested within the randomly selected health facilities. The second level explored variability between facilities in job satisfaction and job conditions reported by nurses.

Initially exploratory factor analysis was performed to examine patterns among the job condition items and created subscales, but the analysis did not show meaningful item classifications. We used alternative approaches to classify the job condition items into intrinsic and extrinsic motivational factors subscales, based on expert opinion and published articles [8, 34–36]. Twelve items were classified as intrinsic motivational factors, and they were grouped into 3 subscales: recognition (3 items), professional development (3 items), and features of the work itself (6 items). The other 22 items were classified as extrinsic motivational factors, and they were grouped into 5 subscales: remuneration (3 items), supervision (4 items), interpersonal relationship (3 items), work conditions (8 items), and living conditions (4 items). We combined the scores of the 5-point Likert scale items for each subscale and computed composite mean scores, ranging from 1 to 5.

Cronbach’s Alpha coefficient and average correlation between items were calculated to assess the reliability of items for each subscale. The coefficient has an acceptable value of 0 to 1; 0.7 or higher is a benchmark for items’ internal consistency, but in empirical studies a value of 0.6 or above is acceptable [37].

The key outcome variable was overall job satisfaction. Nurses’ responses were coded into dichotomous levels – with 1 = satisfied (combining responses of strongly agree and agree) and 0 = not satisfied (combining responses of neutral, strongly disagree, and disagree) – in order to improve statistical power for performing group comparisons and statistical tests; and to contribute to meaningful interpretation and presentation of results. Each job condition item was similarly coded into 1 (satisfied) and 0 (not satisfied).

Chi-square tests were performed to examine whether differences in overall job satisfaction by nurses’ characteristics were statistically significant after accounting for clustering effects. The relationship between dichotomous predictors (each job condition item, coded as 1 = satisfied; 0 = not satisfied) and the outcome variable (overall job satisfaction, coded as 1 = satisfied; 0 = not satisfied) was assessed using Tetrachoric correlation coefficient (TCC), which shows strengths of degree of relationship between intrinsic and extrinsic motivation items with overall job satisfaction [38]. Thus TCC is an important indicator to guide policy makers to prioritize interventions in order to increase nurse’s job satisfaction in limited resource settings. Independent sample t-tests and ANOVA were used to examine associations between nurses’ characteristics (sex, educational qualifications, facility type, and work experience) and composite mean scores on the intrinsic and extrinsic motivation subscales.

Multilevel bivariate logistic regression models were fitted to investigate the independent contributions of predictors to the outcome (overall job satisfaction). First, the null model with the outcome variable but without predictors was fitted to calculate the Intraclass Correlation Coefficient (ICC). The ICC is an indication of the need for multilevel analysis and shows the degree of dependency within two randomly selected nurses belonging to the same health facility and thus sharing similar facility characteristics [39, 40]. The ICC ranges from a minimum of 0 if there is no correlation among responses from the same facility, suggesting that clustering is irrelevant, to a maximum of 1 if all responses from the same facility are identical, suggesting that clustering is important. Research shows that ICC generally does not exceed 0.20 for cross-sectional studies [40].

Multilevel multivariable logistic regression models were then fitted with combinations of nurses’ characteristics (sex, age, service years, educational qualification and type of health facility) and intrinsic and extrinsic motivation subscales to assess their independent effects on overall job satisfaction while controlling for other variables. Multicollinearity among predictors was checked before fitting the final model. Candidate predictors from the bivariate model were included in the multivariable model if their *p* value ≤0.25. Adjusted odds ratios (AORs) with 95% confidence intervals were performed. A *p*-value < 0.05 and 95% confidence intervals were considered for statistical significant.

### Scale reliability and variation

Results of exploratory data analysis show that the Cronbach alpha coefficient for all job conditions was 0.89, with an average inter-item correlation of 0.20. Intrinsic and extrinsic motivational factors had Cronbach alpha coefficients of 0.80 and 0.84, respectively, suggesting that the measurement tools were internally consistent (Table 1).

**Table 1:** Item reliability and intraclass correlation coefficients

Category	Number of items	Cronbach alpha coefficient	Average inter-item correlations	ICC between facilities	ICC within facility
All job conditions	**34**	**0.89**	**0.20**	**0.19**	**0.81**
Intrinsic motivational factors	**12**	**0.80**	**0.24**	**0.22**	**0.78**
Recognition	3	0.48	0.23	–	–
Opportunity for advancement	3	0.62	0.35	–	–
Features of the work itself	6	0.61	0.21	–	–
Extrinsic motivational factors	**22**	**0.84**	**0.19**	**0.29**	**0.71**
Remuneration	3	0.67	0.41	–	–
Supervision	4	0.83	0.55	–	–
Interpersonal relationships	3	0.63	0.36	–	–
Working conditions	8	0.73	0.23	–	–
Living conditions	4	0.66	0.33	–	–

The ICC values indicate that 22% of the total variation in responses to intrinsic motivational items reflected differences between facilities, while 78% reflected differences between nurses within facilities. Clustering by facility accounted for slightly more of the total variation in responses to extrinsic motivational items (29%) (Table 1).

### Ethical considerations

Ethical approval was obtained from the Johns Hopkins School of Public Health Institutional Review Board (JHSPH IRB) (reference number 0005303), which met the criteria for exemption under 45 CFR 46.101(b), Category (5). In Ethiopia, we did not ask the National Research Ethics Review Committee (NRERC) to obtain ethical approval/waiver for the following two reasons: a) This study had no significant risks on study participants as approved by JHSPH IRB; b) NRERC is primarily focused on providing ethical decisions for clinical trials involving new drugs, experimental research and studies that require human biological specimens/samples. Instead of NRERC ethical approval, the human resources for health experts at FMoH reviewed the study protocol and granted permission to conduct the study. Verbal consent was obtained from each study participant as approved by JHSPH IRB. All study participants were received information on the study objectives and recruitment process. To protect participants from risks, the study did not record participants’ names, identification numbers and names of health facilities where he or she worked. Data collector interviewed study participant in a private room without disrupting patient care and other health service activities. After completing interview, data collectors put questionnaires in a sealed envelope to keep all answers confidential to anyone at the sample health facility or any other health facility. Data were also entered in a computer with unique random generated ID numbers given for each study participant.

## Results

### Characteristics of study participants and level of job satisfaction

Of the 424 nurses who participated in the study, 390 worked at health centers and 34 at hospitals. Over half of respondents (52.8%) were female, 43.9% were between 25 to 29 years of age, and 61.8% had less than 5 years of work experience. A large majority (91.9%) of nurses worked at health centers, and 86.6% held a diploma. Overall, 60.8% (95% CI = 56.0, 65.5%) of nurses said they were satisfied with their jobs. A greater proportion of female than male nurses expressed satisfaction with their current jobs (65.6% [95% CI = 59.0, 71.8%] versus 55.5% [95% CI = 48.3, 62.5%], *p* = 0.04). Job satisfaction levels were significantly higher for nurses older than 29 years (67.8% [95% CI = 56.7, 77.3%], *p* = 0.048) and those with more than 10 years of work experience (68.8% [95% CI = 55.7, 80.1%], *p* = 0.007) (Table 2).

**Table 2:** Personal characteristics of nurses and overall job satisfaction, by socio-demographic and employment characteristics

Characteristic	Distribution of nurses		Percent of nurses who are satisfied with their job	
	Number	Percent	Percent [95% C.I]	p Pearson Chi-square
Total	424	100	60.8 [56.0,65.5]	
Sex				
Male	200	47.2	55.5 [48.3,62.5]	**0.040**
Female	224	52.8	65.6 [59.0,71.8]	
Age				
< 25 years	123	29.0	65.0 [55.8,73.2]	**0.048**
25 to 29 years	186	43.9	53.8 [45.6,61.7]	
≥ 30 years	115	27.1	67.8 [56.7,77.3]	
Educational qualification				
BSc	57	13.4	56.1 [42.4,69.2]	0.411
Diploma/TVET	367	86.6	61.6 [49.3,70.1]	
Years of service in public health sector				
< 5 years	262	61.8	63.7 [57.6,69.6]	**0.007**
5 to < 10 years	101	23.8	48.5 [38.4,58.7]	
10 years or more	61	14.4	68.8 [55.7,80.1]	
Type of facility				
Hospital	34	8.1	61.8 [43.6,77.8]	0.910
Health center	390	91.9	60.8 [55.7,65.6]	
Region				
Tigray	35	8.3	88.6 [73.3,96.8]	**< 0.001**
Amhara	116	27.4	75.0 [66.1,82.6]	
Oromia	145	34.2	42.8 [34.6,51.2]	
SNNP	88	20.6	62.5 [51.5,72.6]	
City administrations/urban ^a^	14	3.4	60.0 [53.3,64.0]	
Developing regions ^b^	26	6.1	42.7 [34.0,49.7]	

Note: ^a^ includes Addis Ababa, Dire Dawa, Harari; ^b^ includes Afar, Somali, Gambela, Benshangul-Gumuz regions

### Satisfaction with items related to intrinsic motivation

The proportion of nurses who were satisfied with job conditions related to intrinsic motivation was highest for community recognition (93%), features of the work itself (ranging from 60.9 to 77.9%), and access to coaching and mentoring (62%). The proportion was lowest for the availability of opportunities for promotion (28.3%) and receiving needed training (39.6%). There was no significant difference in satisfaction by facility type except for work load (61.8% for hospitals and 78.5% for health centers; *p* < 0.05). Every item was associated with overall job satisfaction, but the strongest relationships were for “I feel that the organization values my work” (TCC = 0.51) and “I received recognition for doing good work” (TCC = 0.56) (Table 3).

**Table 3:** Percent of nurses who were satisfied with job conditions related to intrinsic motivation, by facility type, and association with overall job satisfaction

Item	% of nurses who were satisfied			Association with overall job satisfaction (TCC)^a^
	Hospitals (*n* = 34)	Health centers (*n* = 390)	All nurses (*n* = 424)	
Recognition				
I feel that the organization values my work	50.0	49.7	49.8	0.51*
I received recognition for doing good work	50.0	58.2	57.6	0.56*
I feel that the community values my work	91.2	93.6	93.4	0.32*
Opportunity for advancement				
I feel there are sufficient opportunities for promotion with my employer	20.6	29.0	28.3	0.44*
I have been given the training that I need to succeed in my position	38.2	39.7	39.6	0.38*
I have access to coaching and mentoring to improve my skills when needed	64.7	61.8	62.0	0.40*
Features of the work itself				
My work load is reasonable	61.8*	78.5*	77.1	0.48*
I can take time to eat lunch almost every day	58.8	61.0	60.9	0.27*
The job is a good match for my skills and experience	82.4	69.0	70.1	0.44*
My job description is clear and up to date	68.7	71.3	71.0	0.39*
I am not worried about losing my job	70.6	78.6	77.9	0.34*
My annual performance appraisal is based on my work plan	64.7	75.1	74.3	0.49*

*p < 0.05; ^a^ Tetrachoric correlation coefficient (TCC)

### Satisfaction with items related to extrinsic motivation

The proportion of nurses who were satisfied was extremely low for all items related to remuneration (ranging from 11.6 to 28.6%), moderate for supervision items (54.7 to 68.6%) and consistently high for items related to interpersonal relationships (78.3 to 97.2%). There were significant differences by facility type for fairness of salary compared to other staff, development of work plan with supervisor, and access to electricity and water. Most items were associated with overall job satisfaction; the strongest relationships were for “My supervisor applies personal policies and practices fairly to me” (TCC = 0.55) and “My salary package is fair” (TCC = 0.60). (Table 4).

**Table 4:** Percentage of nurses who were satisfied with extrinsic motivational items, by facility type, and association with overall job satisfaction

Item	% of nurses who were satisfied			Association with overall job satisfaction (TCC)^a^
	Hospitals (*n* = 34)	Health centers (*n* = 390)	All nurses (*n* = 424)	
Remuneration				
My salary package is fair	5.9	12.1	11.6	0.60*
My salary is fair compared to other staff with the same level of responsibility	14.7*	29.7*	28.6	0.36*
My benefits like transport, housing and duty allowance, etc., are fair compared to other staff at my level	14.7	12.1	12.3	0.41*
Supervision				
My supervisor applies personal policies and practices fairly to me	64.7	53.9	54.7	0.55*
My supervisor is available when I need support	64.7	58.5	59.0	0.46*
I have a work plan developed with my supervisor	76.5*	67.9*	68.6	0.41*
The head of this health facility is competent	67.7	65.9	66.0	0.38*
Interpersonal relationships				
I have good relationships with co-workers	100	96.9	97.2	0.12
The morale level in my workgroup is good	76.5	78.5	78.3	0.41*
I consider myself a part of the local community that I served as a health worker	97.1	95.9	96.0	0.36*
Working conditions				
At work, I have good access to electricity	93.9*	61.5*	64.1	0.08
At work, I have access to safe clean water	73.5*	40.7*	43.4	0.12
I have safe and efficient transportation to work	15.6	9.3	9.8	0.15
I have the supplies I need to do my job well	76.5	64.6	65.6	0.45*
I have the working equipment I need to do my job well and efficiently	58.8	15,9	19.6	0.25*
The facility has good access to drugs and medication	64.7	52.6	53.6	0.42*
My workplace is clean	61.8	52.6	53.3	0.38*
The facility takes specific measures to protect me against HIV/AIDs and other occupational hazards	41.2	43.3	43.2	0.40*
Living conditions				
At home, I have good access to electricity	73.5	63.7	64.5	0.13
At home, I have access to safe clean water	67.7	47.2	48.8	0.17
The community where I live has good shopping and entertainment	26.5	10.6	11.9	0.26
I have access to good schooling for my children	56.0	23.3	26.5	0.34*

*p < 0.05; ^a^ Tetrachoric correlation coefficient (TCC)

### Relationship between motivational subscales and nurses’ characteristics

Composite mean scores for intrinsic and extrinsic motivational factors were 3.5 and 3.0, respectively. Mean scores on intrinsic motivational subscales ranged from 2.9 (opportunities for development to 3.7 (features of the work itself). Mean scores on extrinsic motivational subscales ranged from 2.1 (for remuneration) to 4.4 (for interpersonal relationships) (Table 5).

**Table 5:** Mean scores for extrinsic and intrinsic motivational subscales, by nurses’ characteristics (*N* = 424)

Subscale	Mean score ^a^	Gender		Educational qualification		Facility type		Years of service		
		Male	Female	BSc	Diploma	Hospital	Health center	< 5	5–10	> 10
Intrinsic motivational factors										
Recognition	3.6	3.6	3.7	3.5	3.7	3.7	3.7	3.7	3.6	3.7
Opportunities for advancement	2.9	2.8*	3.0*	2.8	2.9	2.9	2.9	2.7	2.7	2.8
Features of the work itself	3.7	3.6*	3.8*	3.6	3.7	3.6	3.7	3.7	3.6	3.8
*Composite*	3.5	3.4*	3.6*	3.4	3.5	3.5	3.5	3.5	3.4	3.6
Extrinsic motivational factors										
Remuneration	2.1	2.0	2.2	2.1	2.1	1.9	2.1	2.2**	1.9**	2.1**
Supervision	3.4	3.4	3.4	3.3	3.5	3.6	3.4	3.4	3.4	3.5
Interpersonal relationships	4.4	4.3	4.4	4.1*	4.4*	4.4	4.4	4.3	4.4	4.4
Work conditions	2.9	2.8*	3.0*	3.0	2.9	3.4*	2.8*	2.9	2.8	3.00
Living conditions	2.6	2.5	2.6	2.6	2.6	3.4*	2.5*	2.6	2.7	2.7
***Composite***	3.0	2.9*	3.1*	3.0	3.0	3.4*	3.0*	3.0	3.0	3.1

^a^ Mean score range = 1–5

^*^ *p* (Independent sample t-tests) < 0.05; ^**^ *p* (ANOVA) < 0.05

Composite scores for both intrinsic and extrinsic motivational factors were significantly higher for female than male nurses. Female nurses also expressed significantly higher satisfaction with opportunities for development (3.0), features of the work itself (3.8) and work conditions (3.0). The score for remuneration was significantly lower for nurses with 5 to 10 years of service (1.9) than for either less experienced or more experienced colleagues (2.2 and 2.1, respectively). Nurses who worked at hospitals were more satisfied with working and living conditions than nurses who worked at health centers (Table 5).

### Predictors of overall job satisfaction

In the bivariate analysis, all intrinsic and extrinsic motivational factors were associated with overall job satisfaction, as were nurse’s age and years of service. In the multivariable model, years of service remained significant along with four motivational factors. After controlling for other variables, nurses with 5 to 10 years of experience were less likely to be satisfied with their job than nurses with fewer years of experience (AOR = 0.37, 95% CI = 0.17, 0.79). Nurses were more likely to be satisfied with their job if they expressed greater satisfaction with remuneration (AOR = 2.04, 95% CI = 1.36, 3.06), recognition (AOR = 2.21; 95% CI = 1.38, 3.53), professional development (AOR = 1.54; 95% CI = 1.06, 2.29), and features of the work itself (AOR = 1.65; 95% CI = 1.20, 2.91) (Table 6).

**Table 6:** Multilevel logistic regression models

Predictors	Bivariate model		Multivariable model	
	Crude odds ratio (COR)	95% CI	Adjusted odds ratio (AOR)	95% CI
Sex (ref. male)	1			
Female	1.53	0.97, 2.42	1.39	0.79, 2.44
Age (ref. < 25 yrs)	1			
25–29 yrs	0.51	0.29, 0.90*	0.64	0.32, 1.28
≥ 30 yrs	0.83	0.44, 1.56	1.29	0.50, 3.34
Educational qualification (ref. BSc)	1			
Diploma/TVET	1.50	0.78, 2.91	1.58	0.69, 3.59
Years of service (ref. < 5 yrs)	1			
5 to < 10 years	0.51	0.30, 0.88*	0.37	0.17, 0.79*
10 years or more	1.12	0.55, 2.26	0.65	0.22, 1.87
Facility type (ref. Hospital)	1			
Health center	0.84	0.32, 2.25	–	–
Public health service obligation (ref. Yes)	1			
No	0.67	0.33, 1.38	–	–
Motivational factors				
Remuneration	3.04	2.14, 4.33*	2.04	1.36, 3.06*
Supervision	2.63	1.95, 3.54*	1.19	0.83, 1.73
Interpersonal relationship	2.04	1.38, 3.04*	0.79	0.47, 1.32
Work conditions	3.22	2.21, 4.70*	1.24	0.72, 2.14
Living conditions	1.84	1.37, 2.44*	1.31	0.87, 1.97
Recognition	4.01	2.80, 5.76*	2.21	1.38, 3.53*
Opportunities for development	3.19	2.32, 4.37*	1.54	1.06, 2.29*
Features of the work itself	5.36	3.41, 8.41*	1.65	1.20, 2.91*

*p < 0.05-statistically significant

## Discussion

This study found lower job satisfaction and motivation levels among nurses than previous research conducted in low- and middle-income countries, including studies in Nigeria [16], Slovenia [17], Cyprus [18], Ghana [19], Papua New Guinea [20] and one zone of Ethiopia [22]. There is a compelling need for policy makers to devise and institutionalize mechanisms to improve job satisfaction among public sector nurses in Ethiopia, based on the predictors of job satisfaction identified in this study.

Nurses with 5 to 10 years of service were less likely to be satisfied with their jobs than nurses with either less or more experience, which is consistent with previous studies in Slovenia [17], Cyprus [18] and South Africa [41]. One possible explanation is that nurses in Ethiopia – who are required to provide compulsory service for up to 5 years after graduation – may have higher expectations regarding pay, benefits, and continuing professional development once their compulsory service period is over; thus, they are likely to be dissatisfied if these expectations are not met. This should be a consideration for policy makers to design retention strategies for nurses; there should be an emphasis on satisfying and motivating nurses who complete their compulsory service.

The literature shows that recognition, professional advancement, and features of the work itself enhance motivation, job satisfaction, and retention among health workers in low- and middle-income countries [13–15, 22, 36, 41–43]. This study confirms that these three intrinsic motivational factors are strong predictors of overall job satisfaction. In this study, nurses’ perceptions of organizational recognition were much less positive than their perceptions of community recognition. This suggests that creating mechanisms for facilities, supervisors, and colleagues to recognize nurses who perform well would be an inexpensive, yet effective way to increase job satisfaction and motivation; and improving health system performance [4, 43, 44]. Similarly, nurses’ perceptions of opportunities for training and promotion were more negative than their perceptions of onsite coaching and mentoring, suggesting that policy makers could improve motivation by expanding limited opportunities for training and creating pathways for promotion.

Remuneration (including salary and fringe benefits) was the only extrinsic motivational factor associated with overall job satisfaction and it received a very low score, suggesting that the salary and benefits package is an important source of dissatisfaction for nurses. This is consistent with the literature [15, 16, 20, 26, 41, 45–49]. WHO [50] has concluded that low salaries may discourage people from entering health care professions and lead to dissatisfaction and poor motivation among existing health workers. In January 2017, the Ethiopian government increased salary for all public servants, including health care professionals, which may help address this critical issue. However, managers in the health sector, including facility managers, must ensure that salary increases are appropriately distributed and uniformly applied, given the perceived lack of fairness around salary. To address these perceptions, policy makers and health managers should also review and revisit human resource management (HRM) policies and improve awareness about pay scales and benefits across health workers with similar responsibilities.

Facility type was not a significant predictor of job satisfaction, but nurses’ perceptions of working and living conditions were significantly worse at health centers than hospitals, likely because health centers often lack essential supplies and equipment and are located in areas with limited access to good schooling for children, electricity, and clean water [2]. Given that most public sector nurses in Ethiopia are posted at health centers, policy makers should make an extra effort to provide a conducive work environment at health centers, including essential supplies and equipment, in order to satisfy, motivate and retain nurses in the public health care system.

WHO has recommended implementing a bundle of human resources management policies to improve health worker motivation, satisfaction, retention, and performance, which, in turn, may help health systems attain high and effective service coverage. Recommended policies provide for: job security, manageable workload, supportive supervision, opportunities for continuing education and professional development, enhanced career development pathways, incentives (e.g., hardship, housing, and education allowances), adequate facilities and working supplies, and improved occupational health and safety [5, 15, 16, 21, 51]. The International Council of Nurses [51] also suggests that remuneration alone (basic salary plus incentives) is not sufficient to retain, satisfy, and motivate health professionals. It must be combined with fair, equitable and transparent non-financial rewards like recognition for achievements, career and professional development and workload management.

Human resource management structure, capacity and practices are weak in Ethiopia, which contributes to low satisfaction, poor motivation, poor working conditions and high intention to leave the job [2, 6, 30, 52]. The FMoH is trying to address mechanisms within its five-year (2016–2020) health sector transformation plan and ten year (2016–2025) HRH strategic plan to improve health care professionals’ including nurses’ motivation, satisfaction and performance [2, 6]. Therefore, our findings guide the FMoH to provide holistic HRM interventions for attracting new graduates and retaining existing nursing staff to serve in primary health care units especially in remote and rural areas.

### Strengths and limitations

We believe this study to be the first nationally representative investigation of nurses’ job satisfaction and motivation in Ethiopia’s public health facilities. The study employed a multilevel analysis to obtain precise estimates after adjusting for clustering effects. Thus, the study has power to generalize findings to the country and can be applied in low and middle-income countries to develop human resources for health retention strategy, including the nursing workforce. Although we did not triangulate with qualitative findings, we feel the data are comprehensive and sufficient to understand the situation with regard to nurses’ job satisfaction and motivation in the country. There are some limitations related to the sampling. The final sample size of 424 was less than expected 500 nurses, despite the provision made for a 10% non-response rate. Lack of evidence on study tool reliability and validity was also a limitation.

### Contributions of the study findings to the local and global nursing communities

The study highlighted the national level of nurses’ job satisfaction and motivation; and its associated factors for the low income country-Ethiopia. The study findings inform the Ethiopian Ministry of Health, regional health bureaus, Ethiopian Nursing Association and other stakeholders who invest in the Ethiopian health sector to plan appropriate interventions that promote nurse retention within the public health sectors. The findings can also help other low and middle income countries to design appropriate nursing workforce retention strategy, especially for increasing availablity and competent nursing professionals to meet the aggregate density of 4.45 doctors, nurses and midwives per 1000 population by 2030 [5]. Our study will also provide unique contributions to the existing body of literature on nurses’ job satisfaction and motivation globally for the following reasons: a) the study used standard and rigorous research methods; employed a large sample size and was designed meticulously to provide credible nationally representative information for Ethiopia. It includes randomly selected hospitals and health centers located in rural and remote areas as well as urban areas. The study represents findings of a low income country where national studies of this scale are rarely conducted, thus adding a new perspective with precise estimates on nurses’ job satisfaction and motivation to the global literature could be helpful; b) we analyzed a combination of intrinsic and extrinsic motivational factors instead of specific job items in order to explore potential factors that associated with nurses’ job satisfaction. We believe that investigating all job conditions using established theory may guide policy makers and researchers to enhance nurses’ job satisfaction and motivation; and ultimately contributes to enhance nurses’ performance in nursing care quality; c) many studies (e.g. [9, 16, 22, 23, 41]) on this topic used conventional statistical analysis. However, we employed multilevel analysis to account for clustering effects in order to maximize the precision of estimates for making appropriate policies to increasing nurses’ job satisfaction and motivation. Others (students, researchers or policy makers) may learn from our analytical approaches for performing similar studies of job satisfaction and motivation; or similar health-related problems.

## Conclusions

The study findings are signals for the FMoH and Regional Health Bureaus to strengthen the human resource management system and practices to improve nurses’ overall job satisfaction and motivation, especially among nurses with 5–10 years of experience on the job. Expanded recognition systems and opportunities for advancement are needed to increase nurses’ motivation and job satisfaction, while equitable and transparent salary and benefits packages are also needed to reduce their dissatisfaction with the job. The findings may serve as a benchmark for the government’s 10-year HRH strategic plan and to evaluate the effectiveness of various HRM interventions to be implemented from 2016 to 2025. Moreover, the study contributes to low- income countries to enhance performance of nurses and improving quality in nursing care. We recommend conducting a mixture of quantitative and qualitative research to explore reasons for low satisfaction related to remuneration, work conditions and living conditions for three groups of nurses: male nurses, nurses working in health centers, and nurses with 5 to 10 years of working experience.

## Data Availability

The questionnaires and datasets used and/or analyzed during the current study are available from the corresponding author on reasonable request.

## Abbreviations

AOR: Adjusted Odds Ratio
COR: Crude Odds Ratio,
FMoH: Federal Ministry of Health
HRH: Human Resources for Health
HRM: Human Resources Management
ICC: Intraclass correlation coefficient
JHSPH IRB: Johns Hopkins School of Public Health Institutional Review Board
NRERC: National Research Ethics Review Committee
SDG: Sustainable Development Goal
SNNP: Southern Nations Nationalities and Peoples
TCC: Tetrachoric correlation coefficient
TVET: technical and vocational education and training
USAID: United States Agency for International Development
WHO: World Health Organization

## References

[1] World health statistics 2018: monitoring health for the SDGs, sustainable development goals. Geneva: World Health Organization; 2018. http://apps.who.int/iris/bitstream/handle/10665/272596/9789241565585-eng.pdf?ua=1. Accessed 28 Aug 2018.

[2] Federal Democratic Republic of Ethiopia Ministry of Health. Health sector transformation plan: 2015/16–2019/20. Addis Ababa: Ethiopia Ministry of Health; 2015. http://www.moh.gov.et/documents/26765/0/Health+Sector+Transformation+Plan/5542a23a-9bc7-46a2-8c1f-8b32c2603208?version=1.0. Accessed 7 Mar 2017.

[3] Central Statistical Agency (CSA) [Ethiopia] and ICF. 2016. Ethiopia Demographic and Health Survey 2016. Addis Ababa, Ethiopia, and Rockville, Maryland, USA: CSA and ICF. https://dhsprogram.com/pubs/pdf/FR328/FR328.pdf. Accessed 28 Aug 2018.

[4] World Health Organization. Everybody’s business: strengthening health systems to improve health outcomes: WHO’s framework for action. Geneva: WHO; 2007. http://www.who.int/healthsystems/strategy/everybodys_business.pdf. Accessed 14 June 2017.

[5] World Health Organization. Global strategy on human resources for health: workforce 2030. Geneva: World Health Organization; 2016. http://apps.who.int/iris/bitstream/10665/250368/1/9789241511131-eng.pdf?ua=1. Accessed 25 Feb 2017.

[6] Federal Democratic Republic of Ethiopia Ministry of Health. National human resources for health strategic plan 2016-2025, Addis Ababa, September 2016. Policy document.

[7] Kenya Ministry of Health, Nursing Council of Kenya, Emory University, U.S. Centers for Disease Control and Prevention. Kenya Nursing Workforce Report: The Status of Nursing in Kenya, 2012. http://www.health.go.ke/wpcontent/uploads/2015/09/Kenya%20Nursing%20Workforce%20Report.pdf. Accessed 28 May 2017.

[8] Armstrong M (2009). Armstrong’s handbook of human resource management practice.

[9] Bonenberger M, Aikins M, Akweongo P, Wyss K (2014). The effects of health worker motivation and job satisfaction on turnover intention in Ghana: a cross-sectional study. Hum Resour Health.

[10] Lu H, While AE, Barribal KL (2005). Job satisfaction among nurses: a literature review. Int J Nurs Stud.

[11] Twigg D, McCullough K (2014). Nurse retention: a review of strategies to create and enhance positive practice environments in clinical settings. Int J Nurs Stud.

[12] Cicolini G, Comparcini D, Simonetti V (2014). Workplace empowerment and nurses’ job satisfaction: a systematic literature review. J Nurs Manag.

[13] Halcomb E, Smyth E, McInnes S. Job satisfaction and career intentions of registered nurses in primary health care: an integrative review. BMC Family Practice (2018)19:136. 10.1186/s12875-018-0819-1 PMC6081816 30086722

[14] Dormon F, Balen J, Schmidtke KA, Vlaev I. Health workers’ motivation in low and middle income countries: A systematic review of the literature. Medical Research Archives. Volume 5, issue 8. 2017.

[15] Buchan J, Shaffer FA, Catton H. Policy Brief. Nurse Retention. 2018. International Center on Nurse Migration. https://www.icn.ch/sites/default/files/inline-files/2018_ICNM%20Nurse%20retention.pdf . Accessed 8 July 2018.

[16] Chirdan OO, Akosu JT, Ejembi CL, Bassi AP, Zoakah AI (2009). Perceptions of working conditions amongst health workers in state-owned facilities in northeastern Nigeria. Ann Afr Med.

[17] Lorber M, Skela SB (2012). Job satisfaction of nurses and identifying factors of job satisfaction in Slovenian hospitals. Croat Med J.

[18] Lambrou P, Kontodimopoulos N, Niakas D (2010). Motivation and job satisfaction among medical and nursing staff in a Cyprus public general hospital. Hum Resour Health.

[19] Bempah OSB (2013). Determinants of job satisfaction among community health workers in the Volta region of Ghana. Pub Policy Adm Res.

[20] Jayasuriya R, Whittaker M, Halim G, Matineau T (2012). Rural health workers and their work environment: the role of inter-personal factors on job satisfaction of nurses in rural Papua New Guinea. BMC Health Serv Res.

[21] World Health Organization. Increasing access to health workers in remote and rural areas through improved retention: global policy recommendations. Geneva: WHO; 2010. http://www.who.int/hrh/retention/guidelines/en/. Accessed 30 Jan 2017. 23741785

[22] Semachew A, Belachew T, Temamen Tesfaye T, Mehretie Adinew YM (2017). Predictors of job satisfaction among nurses working in Ethiopian public hospitals, 2014: institution-based cross-sectional study. Hum Resources for Health.

[23] Asegid A, Belachew T, Yimam E. Factors influencing job satisfaction and anticipated turnover among nurses in Sidama Zone public health facilities, South Ethiopia. Nurs Res Pract. 2014. 10.1155/2014/909768. 10.1155/2014/909768 PMC3953615 24707397

[24] Salilih SZ, Abajobir AA (2014). Work-related stress and associated factors among nurses working in public hospitals of Addis Ababa, Ethiopia: a cross-sectional study. Workplace Health Saf.

[25] Mengistu MM, Bali GA (2015). Factors associated to job satisfaction among healthcare workers at public hospitals of west Shoa zone, Oromia regional state, Ethiopia: a cross sectional study. Sci J Public Health.

[26] Hotchkiss DR, Hailom Banteyerga H, Manisha TM (2015). Job satisfaction and motivation among public sector health workers: evidence from Ethiopia. Hum Resour Health.

[27] Getie GA, Betre ET, Hareri HA (2015). Assessment of factors affecting turnover intention among nurses working at governmental health care institutions in east Gojjam, Amhara region, Ethiopia, 2013. Am J Nurs Sci.

[28] Engeda EH, Birhanu AM, Alene KA (2014). Intent to stay in the nursing profession and associated factors among nurses working in Amhara regional state referral hospitals. Ethiopia BMC Nurs.

[29] Dagne T, Beyene W, Berhanu N (2015). Motivation and factors affecting it among health professionals in the public hospitals, Central Ethiopia. Ethiop J Health Sci.

[30] Ayalew F, Kols A, Kim YM, Schuster A, Emerson MR, Roosmalen JV (2015). Factors affecting turnover intention among nurses in Ethiopia. World Health Popul.

[31] Federal Democratic Republic of Ethiopia Ministry of Health. Health and Health Related Indicators 2005 E. C (2012/2013). Version 2, Oct 2014. https://www.dktethiopia.org/sites/default/files/PublicationFiles/Health%20and%20Health%20Related%20Indicators%202005%20E.C.pdf. Accessed 5 Jan 2017.

[32] Sampling Manual for Facility Surveys for Population, Maternal Health, Child Health and STD Programs in Developing Countries. MEASURE Evaluation Manual Series, No. 3. MEASURE *Evaluation*. Carolina Population Center, University of North Carolina at Chapel Hill. July 2001. http://www.cpc.unc.edu/measure/publications/ms-01-03. Accessed 17 March 2017.

[33] Capacity Project (2006). Job satisfaction survey (draft).

[34] Willis-Shattuck WM, Bidwell P, Thomas S, Wyness L, Blaauw D, Ditlopo P (2008). Motivation and retention of health workers in developing countries: a systematic review. BMC Health Serv Res.

[35] Alpern R, Canavan ME, Thompson JT, McNatt Z, Tatek D (2013). Development of a brief instrument for assessing healthcare employee satisfaction in a low-income setting. PLoS One.

[36] Vigan FA, Giauque D. Job satisfaction in African public administrations: a systematic review. Int Rev Adm Sci. 2016. 10.1177/0020852316651693.

[37] Erik M, Marko S (2011). A concise guide to market research, the process, data, and methods using IBM SPSS statistics.

[38] Bonnet DG, Price RM (2005). Inferential methods for the tetrachoric correlation coefficient. J Educ Behav Stats.

[39] Snijder TAB, Bosker RJ (2003). Multilevel analysis: an introduction to basic and advanced multilevel modeling.

[40] Twisk JWR (2010). Applied multilevel analysis: a practical guide.

[41] Pillay R (2009). Work satisfaction of professional nurses in South Africa: a comparative analysis of the public and private sectors. Hum Resour Health.

[42] Lakra GJ, Kadam S, Hussain MA, Pati S, Sharma K, Zodpey S (2012). Motivation and job satisfaction among multipurpose health workers in hilly and non-hilly areas of Jashpur District, Chhattisgarh: an exploratory study. Southeast Asian J Trop Med Public Health.

[43] Lefton C (2012). Strengthening the workforce through meaningful recognition. Nurs Econ.

[44] Kumar R, Ahmed J, Shaikh BT, Hafeez R, Hafeez A (2013). Job satisfaction among public health professionals working in public sector: a cross sectional study from Pakistan. Hum Resour Health.

[45] Sansoni J, De Caro W, Marucci AR, Sorrentino M, Mayner L, Lancia L (2016). Nurses’ job satisfaction: an Italian study. Ann Ig.

[46] Schmiedeknecht K, Perera M, Schell E, Jere J, Geoffroy E, Rankin S (2015). Predictors of workforce retention among Malawian nurse graduates of a scholarship program: a mixed-methods study. Glob Health Sci Pract.

[47] McHugh MD, Ma C. Wage, work environment, and staffing: effects on nurse outcomes. Policy Polit Nurs Pract 2014;15(0):72–80. 10.1177/1527154414546868 PMC4667784 25121923

[48] Ugwa E, Charity U (2016). A narrative review of factors affecting job satisfaction among nurses in Africa. Hosp Pract Res.

[49] Bossert T, Bärnighausen T, Bowser D, Mitchell A, Gedik G. Assessing financing, education, management and policy context for strategic planning of human resources for health. WHO 2007. http://www.who.int/hrh/tools/assessing_financing.pdf*.* Accessed 15 Jan 2017.

[50] World Health Organization and Global Health Workforce Alliance. What countries can do now: twenty-nine actions to scale-up and improve the health workforce. Geneva: WHO; 2009. http://www.who.int/workforcealliance/knowledge/toolkit/13.pdf?ua=1. Accessed 6 Mar 2017.

[51] International Council of Nurses, International Hospital Federation, International Pharmaceutical Federation, World Confederation for Physical Therapy, World Dental Federation, World Medical Association. Guidelines: incentives for health professionals. 2008.http://www.who.int/workforcealliance/documents/Incentives_Guidelines%20EN.pdf. Accessed 8 Mar 2017.

[52] Kols A, Kibwana S, Molla Y, Ayalew F, Teshome M, van Roosmalen J, Stekelenburg J. Factors predicting Ethiopian Anesthetists' intention to leave their job. World J Surg. 2017;6. 10.1007/s00268-017-4318-7. 10.1007/s00268-017-4318-7 PMC5895675 29110158

